# Changing the double-pigtail stent by a new suture stent to improve patient’s quality of life: a prospective study

**DOI:** 10.1007/s00345-014-1394-2

**Published:** 2014-09-12

**Authors:** Benoît Vogt, Arnaud Desgrippes, François-Noël Desfemmes

**Affiliations:** Department of Urology, Polyclinique de Blois, 1 rue Robert Debré, 41260 La Chaussée Saint-Victor, France

**Keywords:** Stent, Quality of life, Suture, Thread, Dilation, Ureter

## Abstract

**Purpose:**

Double-pigtail stent intolerance reduces patient’s quality of life. By decreasing the amount of material within the bladder, it should be possible to attenuate stent’s symptoms. We evaluated the tolerance of a new stent with a dedicated questionnaire.

**Methods:**

The major innovation of the pigtail suture stent (PSS) is in the replacement of the lower part of the double-pigtail stent with a 0.3F suture. A total of 79 consecutive patients agreed to be fitted with a PSS. The double-pigtail stents of 24 patients complaining strongly of symptoms were replaced with PSS (group 1), and 55 other patients were fitted directly with the PSS after an ureteral endoscopic intervention (group 2). The questionnaire was prospectively administered to patients at baseline and Day 15 post-placement.

**Results:**

All questionnaires were returned. In group 1, the replacement of the double-pigtail stent with a PSS significantly decreased urinary symptom scores (35.2 ± 7.5 vs. 23.6 ± 5.4; *p* = 2 × 10^−6^) and pain scores (11.0 ± 3.9 vs. 4.9 ± 3.1; *p* = 1 × 10^−7^). In group 1, the baseline scores were not significantly different from those of control group with double-pigtail stent. In group 2, the urinary scores with PSS were significantly different from those of baseline without stent. The scores of the two groups fitted with a PSS were not significantly different at Day 15 post-placement. Unexpectedly, following PSS implantation, we observe a clear dilation of the ureter without inflammation around the suture.

**Conclusions:**

The PSS significantly decreases stent’s symptoms and constitutes a medical advance in the domain of ureteral stent tolerance.

**Electronic supplementary material:**

The online version of this article (doi:10.1007/s00345-014-1394-2) contains supplementary material, which is available to authorized users.

## Introduction

Double-pigtail stents are frequently implanted in the ureter in urological practice. However, they are poorly tolerated, severely impairing the quality of life of patients [1, 2]. Several studies have clearly described symptoms associated with their use: urinary frequency, urgency, dysuria, incontinence, hematuria, incomplete emptying, a feeling of pelvic heaviness, and lumbar pain. These symptoms are due largely to the bladder irritation caused by the stent [3]. It has been suggested that changes to the size, form, and composition of stents could decrease discomfort. Indeed, by decreasing the amount of material in the bladder, it may be possible to attenuate the symptoms [3].

**Fig. 1:**
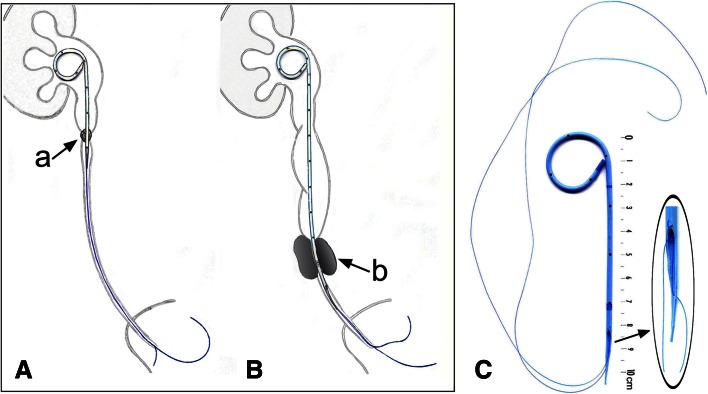
Anatomical position and manually cut of the PSS. **A** Short PSS implanted for a ureteral obstruction, with stent prolongation by a bladder suture. **B** Long PSS for lumbar-iliac or obstructive upper pelvic ureteral stenosis or stone, with intubation of the obstruction by the stent and then by bladder suture prolongation. *a* Obstruction by stone in the ureter or at the ureteropelvic junction. *b* Obstruction due to a stone or a compressive tumor in the ureter. **C** The characteristic innovation of this stent is that the lower part of the stent was replaced with a 0.3F suture. The thinning out of the lower end appears to limit the catching of the stent on the ureter during breathing movements. Without suture, the short model is 10 cm long and the long model is 20 cm long

We developed a pigtail suture stent (PSS), which has been used since December 2010 in 295 patients. In this innovative PSS, the lower part of the stent is replaced by a 0.3F thread of suture. Only the renal and ureteral parts of the stent are retained and are extended by a thin tail ending in a suture (Fig. 1). Whether the obstruction is due to a stone, an ureteropelvic junction syndrome, or ureteral stenosis, the upper, unmodified part of the stent facilitates the passage of urine around the obstacle.

In a previous retrospective study with 56 patients fitted with a non-profiled PSS, we found that tolerance was improved, with surprisingly good levels of bladder tolerance, but frequent anterior flank discomfort. This discomfort seemed to be due to irritation caused by the lower part of the stent, which was sectioned manually and was unmodified. The answer rate to the questionnaires was 75 % [4]. In December 2012, the lower end of the stent was sculpted and thinned, like a radical or rootlet, to prevent irritation of the ureter. This clearly increased PSS tolerance. Furthermore, we discovered fortuitously that PSS had other surprising properties. We observed clear dilation of the ureter intubated with the sutures.

In this study, we evaluate the effect of the PSS with the innovative profiled tail on urinary symptoms and pain by using a dedicated questionnaire.

## Patients and methods

### Technique: construction, implantation, and ablation of the PSS

The short model: A polyurethane double-pigtail stent (Double loop ureteral stents 7F 26 cm, Coloplast) is sectioned perpendicularly to the main axis, 10 cm from the renal loop. The sectioned part is then cut parallel to the main axis, to give two equal parts, each 3 cm long, to form the tail. At the upper end of the tail, this cutting results in a smooth, lateral beveled edge. The tail is then thinned out toward the lower end, where its diameter should not exceed 0.5 mm. A polypropylene suture (Ethicon monofilament polypropylene suture; gauge size U.S.P.1; 0.1–0.15 mm; 5–0) perforates the end of the tail and then the stent, above the beveled edge. A knot is tied at the beveled edge, and the tail of the PSS consists of two 0.3F sutures. Each suture is about 20 cm long. A knot was sometimes tied at the lower end of the sutures. This PSS has a total length of 30 cm.

The long model: a polyurethane double-pigtail tumor stent (Double loop ureteral stent Vortek Tumor Stent 7F 26 cm, Coloplast) is sectioned perpendicularly, ensuring that the stent remains long enough to descend 3 cm below the obstructive ureteral stenosis or stone; it is about 20 cm long. A tail is cut, and a suture only 10 cm long is passed through the stent, as described above.

The intervention is carried out under general or regional anesthesia. The pigtail is placed in the kidney, as for a normal double-pigtail stent under direct vision through the cystoscope and fluoroscopic guidance, but a sufficiently long ureteral stent is used to make up for the shortness of the pushing device. A stent (Open-End Flexi-Tip Ureteral Catheter, 5F, 70 cm, Cook Medical) was placed upside down on the wire guide to look like the end of the usual pusher. Approximately 50 cm is needed to push the PSS. The sutures were left in the bladder. PSS was withdrawn under local or general anesthesia, with the aid of flexible cystoscopy and forceps (Karl Storz—Endoskope, Biopsy Forceps, double action jaws, 7F, length 40 cm, 27175A), by simply pulling on the sutures. The knot at the lower end of the sutures could facilitate stent removal.

### Patients and groups

From January to July 2013 in a single institution, 79 consecutive patients agreed to be fitted with the PSS, with the aim of attenuating their urinary symptoms. Twenty-four of these patients complained strongly of symptoms associated with a double-pigtail stent already fitted (Double loop ureteral stents 7F 26 cm; Coloplast) and sought relief from these symptoms. Their stents were replaced with the PSS (group 1). The other 55 patients received a PSS directly after ureteral endoscopy (group 2). Ten patients fitted with double-pigtail stent for pelvic stone were evaluated as control group.

### Questionnaires

A French translation of the ureteral stent symptoms questionnaire was used to evaluate stent tolerance [5, 6]. We have extracted exactly the 11 questions relating to urinary symptoms (U1-11, total score of 11–56). We used five questions relating to pain (P1-3 and P7-8, total score of 2–17) and one question relating to impact on work (W5, score of 1–5). The questionnaire was administered to the 79 patients at baseline and Day 15 post-placement. The questionnaire was administered to the control group at Day 15 post-placement. The questionnaire was administered to the patients of group 1 at baseline with double-pigtail stent and Day 15 after PSS placement. The questionnaire was administered to the patients of group 2 at baseline without stent and Day 15 after PSS placement. Ten patients were evaluated as control group with double-pigtail stent at Day 15 post-placement. The patient has completed the questionnaire. Our questionnaire was approved by French Ethical Committee (IRB registration 00001072).

### Statistical analysis

The data are presented as mean ± SD. Kruskal–Wallis test and *χ* ^2^ test were used to check the comparability of the groups. A paired or two-tailed Student’s *t* test and Fisher’s exact test were used to compare scores between groups. Values of *p* < 0.05 were considered significant.

## Results

All questionnaires were returned. Sixty-four patients (81.0 %) had obstructive ureteral stones, 3 patients (3.8 %) had obstruction of the ureteropelvic junction, and 12 patients (15.2 %) had obstructive stenosis in the iliac or pelvic ureter. Twenty-three patients received the long model of the PSS for malignant obstructive ureteral stenosis or distal stone. Age, sex, weight, height, side, procedures, and cause of the derivation are summarized in Table 1. The three groups were comparable.

**Table 1:** Patient characteristics

	Group 1 (*n* = 24)	Group 2 (*n* = 55)	Control group (*n* = 10)	*p*
	Double-pigtail and PSS	PSS alone	Double-pigtail	
Age (years)				
Mean ± SD	58.9 ± 15.1	60.0 ± 15.0	53.6 ± 13.1	0.44
Male/female	14/10	34/21	7/3	0.82
Weight (kg)				
Mean ± SD	71.2 ± 15.8	77.0 ± 15.2	80.0 ± 15.3	0.27
Height (cm)				
Mean ± SD	165.2 ± 10.9	166.5 ± 9.0	171.1 ± 9.1	0.40
Side (R/L)	16/8	29/26	6/4	0.51
Indication for stenting				
Proximal stone	15	35	0	
Distal stone	3	11	10	
Ureteropelvic junction	1	2	0	
Ureteral stricture	5	7	0	
Procedures				
Stenting alone	6	13	0	
SWL	2	6	0	
Urine alkalinization	1	2	0	
SWL and ureteroscopy	12	12	0	
Ureteroscopy	3	22	10	
Stent removal				
Flexible cystoscopy	4	15	10	
Ureteroscopy	14	31	0	
Laparoscopy	1	2	0	

Extracorporeal shockwave lithotripsy (SWL)

### Technical results

In group 1, the mean duration of stenting before PSS was 17.3 ± 10.2 days. In all cases, the PSS gave effective renal drainage. No difficulty in the placement of the PSS was encountered. No ureteral stricture induced by the PSS was reported in our patients. The PSS was replaced by double-pigtail stent after laparoscopy for ureteropelvic junction. Twelve patients with ureteral stenosis still have implanted PSS. No calcification was observed on the sutures 6 months after stenting. The stent was removed from the other patients, after 58.8 ± 24.6 days.

Forty-nine patients undergoing repeat surgery for endoscopic stone treatment after PSS implantation presented clear dilation of the ureteral meatus and the ureter. This dilatation facilitated the introduction of a 12F rigid ureteroscope or the sheath of a flexible ureteroscope, without the need for further enlargement.

During stent removal, the bladder sutures were found to have migrated into the urethra in 12 men and 1 woman. This trans-sphincter migration of the sutures had no consequences for continence. Three of the PSS had to be withdrawn under ureteroscopy, because the sutures were cut too short and had migrated into the ureter. In these three cases, stent removal through the ureter was easy without the further enlargement of meatus.

### Functional results

The differences in urinary and pain symptoms between groups 1 and 2 are summarized in Table 2. In group 1, scores for urinary symptoms (35.2 ± 7.5 vs. 23.6 ± 5.4; *p* = 2 × 10^−6^) and pain scores (11.0 ± 3.9 vs. 4.9 ± 3.1; *p* = 1 × 10^−7^) were significantly decreased by the replacement of the double-pigtail stent with a PSS (p*). In group 2, the urinary scores were significantly different from those of baseline (p**). At Day 15 post-placement, the urinary and pain scores for the patients with PSS implants in group 2 were not significantly different from those of patients with PSS implants in group 1 (p***). At baseline, the urinary scores and pain scores in group 1 were not significantly different from those of control group (p****).

**Table 2:** Results of questionnaires

	Group 1 (*n* = 24)			Group 2 (*n* = 55)				Control group (*n* = 10)	
	Baseline Double-pigtail	Day 15 PSS after double-pigtail	p*	Baseline No stent	Day 15 PSS	p**	p***	Double-pigtail	p****
*Urinary tract symptoms*									
Frequency	3.7 ± 1.3	2.8 ± 1.1	0.005	1.9 ± 0.9	2.8 ± 1.0	3 × 10^−7^	0.87	4.0 ± 1.2	0.52
Nocturia	3.8 ± 1.1	2.8 ± 1.2	1 × 10^−4^	2.2 ± 0.9	2.9 ± 1.0	3 × 10^−5^	0.52	3.4 ± 1.4	0.45
Urgency	3.2 ± 1.2	2.4 ± 1.0	0.01	1.4 ± 0.8	2.0 ± 1.0	2 × 10^−4^	0.17	3.2 ± 1.3	0.95
Urge incontinence	2.0 ± 0.9	1.8 ± 0.8	0.46	1.1 ± 0.5	1.4 ± 0.8	0.006	0.03	1.5 ± 0.8	0.15
Non-urge incontinence	1.8 ± 0.09	1.1 ± 0.3	0.004	1.2 ± 0.6	1.3 ± 0.8	0.48	0.14	1.5 ± 0.8	0.46
Incomplete emptying	3.0 ± 1.2	1.8 ± 0.9	3 × 10^−5^	1.2 ± 0.6	1.8 ± 1.0	2 × 10^−6^	0.99	2.5 ± 1.2	0.32
Urethral pain	3.9 ± 1.3	1.8 ± 1.1	5 × 10^−8^	1.1 ± 0.3	1.7 ± 0.8	8 × 10^−6^	0.70	3.4 ± 1.1	0.24
Hematuria	2.8 ± 1.4	1.6 ± 0.8	6 × 10^−4^	1.2 ± 0.5	1.7 ± 1.0	7 × 10^−5^	0.49	2.6 ± 1.8	0.81
Hematuria amount	2.1 ± 0.8	1.6 ± 0.8	0.01	1.1 ± 0.4	1.5 ± 0.7	0.001	0.62	2.1 ± 1.4	0.96
Interference in life	3.8 ± 1.0	2.3 ± 0.9	3 × 10^−5^	1.2 ± 0.6	1.7 ± 0.8	6 × 10^−4^	0.02	3.5 ± 1.4	0.57
Quality-of-life impact	5.3 ± 2.0	3.8 ± 1.6	7 × 10^−4^	1.7 ± 1.0	3.1 ± 1.5	2 × 10^−7^	0.08	5.6 ± 1.3	0.54
Total score	35.2 ± 7.5	23.6 ± 5.4	2 × 10^−6^	15.3 ± 5.6	21.9 ± 6.1	5 × 10^−10^	0.24	33.3 ± 7.3	0.50
*Pain*									
Pain	23 (95.8 %)	13 (54.2 %)	0.002		16 (29.1 %)		0.04	9 (90 %)	0.51
Pain while passing urine	12 (50.0 %)	4 (16.7 %)	0.03		2 (3.6 %)		0.07	6 (60.0 %)	0.71
Painkillers	3.6 ± 1.3	2.0 ± 1.2	2 × 10^−6^		1.3 ± 1.9		0.28	2.8 ± 1.8	0.65
Visual analog scale for pain	5.9 ± 2.8	1.8 ± 2.0	3 × 10^−5^		1.7 ± 1.0		0.31	5.1 ± 2.9	0.46
Total score	11.0 ± 3.9	4.9 ± 3.1	1 × 10^−7^		4.0 ± 2.8		0.20	9.9 ± 4.3	0.49
*Effect on work*									
Effect on work	3.5 ± 1.5	1.8 ± 1.0	2 × 10^−5^		1.5 ± 1.0		0.15	3.7 ± 1.3	0.67

For urinary tract symptoms, all questions in USSQ were used

p* for comparisons between PSS and baseline (double-pigtail stent) in group 1

p** for comparisons between PSS and baseline (no stent) in group 2

p*** for comparisons between the two groups, for the PSS

p**** for comparisons between control group and baseline (double-pigtail stent) in group 1

## Discussion

**Fig. 2:**
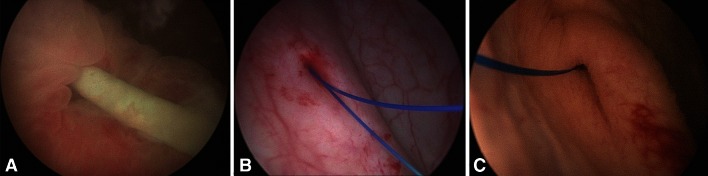
Appearance of the ureteral meatus. **a** Inflamed meatus around the double-pigtail stent. **b** Punctiform meatus immediately after PSS implantation. **c** Dilated meatus 1 month after PSS implantation

Double-pigtail stents are frequently implanted in urological practice, to drain urine. The PSS described here uses the same mode of drainage in patients with obstructions. Whether the obstruction is due to a stone, an ureteropelvic junction syndrome, or ureteral stenosis, the upper, unmodified part of the stent facilitates the passage of urine around the obstacle. In our view, if the obstruction is located in the upper part of the ureter, the rest of the ureter is likely to be healthy and does not require drainage with a stent. The part of the stent in the bladder is, thus, of no use in such conditions, and its presence may provoke secondary effects. It has been suggested that pelvic symptoms could be decreased by reducing the amount of material in the bladder [3]. The replacement of the bladder loop with a fine suture results in the presence of only tiny amounts of material in the bladder. Only the suture should cross the junction between the ureter and the bladder and float in the bladder itself (Fig. 2). The replacement of the lower part of the stent with a suture, resulting in the absence of an internal channel, probably also limits renal reflux.

Stents of several sizes, forms, and compositions have been studied, with the aim of reducing these symptoms. A short bladder loop seems to be preferable to a long loop extending throughout the bladder [7, 8]. The replacement of the bladder loop by a more flexible loop has no effect [9, 10]. Decreasing the diameter of the stent from 6 to 4.8F also has no effect [10]. The beneficial effects or replacing the bladder loop by a collection of loop [3, 11] or by a thinned tail with a diameter of 3F [3, 12] remains a matter of debate.

Joshi et al. [5] obtained a score of 14.9 for the control group without stent. We obtained a score of 15.3 for such patients. Patients with a double-pigtail stent had urinary symptom scores of about 28 [5]. This score was about 30 in a subsequent study of 116 patients [9]. Damiano, Gianarini, and Davenport reported scores of about 27, 30, and 32, respectively [8, 10, 13]. We obtained a score of 35.2 for such patients. The score of our control group is 33.3 and was not significantly different than group 1 at baseline. The PSS decreased the total score from 35.2 to about 23, and this results confirm those of our retrospective study [4]. Few studies have reported a significant decrease in urinary symptoms. In a meta-analysis, Lamb noted that alpha-blockers decreased scores [14]. Kawahara [11] reported that the Polaris Loop^®^ caused fewer symptoms in a group of 25 patients, but this was not confirmed in the series reported by Lingeman [3]. Lee [7] found that correct stent positioning was more important than drug prescription.

The urinary and pain scores linked to the PSS were not significantly different for the patients of the two groups. Thus, the scores for group 1 do not seem to be the consequence of excessive enthusiasm for the PSS following poor tolerance of the double-pigtail stent.

Despite the clear improvement observed with PSS, the patients still had symptoms statistically different from their normal state. It seems that some symptoms decrease with time (dysuria, hematuria), but the general tolerance remains unchanged [15]. However, about 9 months are required to observe a significant decrease in urinary symptoms [16]. Even with PSS, duration of stenting must be as short as possible.

The stent is implanted to ensure the correct drainage of urine. The drainage mediated by the double-pigtail stent has been described previously. In normal ureters, the urine passes between the stent and the ureter wall, rarely through the holes. In ureters that are compromised or have a reduced diameter, the urine passes through the holes and the internal channel. The bladder loop seems to play no role in urine flow [17]. Finally, the diameter of the stent (7 or 3F) has no effect on the efficacy of urine flow [18]. In cases in which the lower ureter is healthy, we can confirm the normal flow of urine around the PSS.

Three of the PSS had to be withdrawn under ureteroscopy, because the sutures were cut too short and had migrated into the ureter. In these three cases, the PSS gave effective renal drainage. Stent removal through the ureter was easy without the further enlargement of meatus. Following these observations, in male patients, we keep a long suture so that the PSS length is 30 cm. In women, we cut the sutures at the urethral meatus. However, we believe that the obstruction must be bypassed by a segment of rigid stent and not by the suture. At the beginning of our experience, we have observed one migration of the suture into the ureter when the obstruction was bypassed by the suture only. If the ureter is healthy, sutures have not been observed to migrate up the ureter.

We felt that it was important to create a device having a perfect profile. It is possible that a straight segment of the ureter better tolerates the stent section and ureteral irritation in the region below the tail induces flank pain. PSS without the profiled tail has been used since December 2010 and has greatly reduced bladder symptoms. But frequent anterior flank discomfort seemed to be due to irritation caused by the lower part of the stent, which was sectioned manually and was unmodified. Because of flank pain, we rarely used this stent for 2 years. In 1994, Ponsot [19] and, in 1995, Dauleh [20] described a new stent prototype respectively in eight and three patients. The lower loop was replaced by a fine strong nylon loop to increase bladder tolerance [19] or prevent natural anti-reflux [20]. But no further study was published.

In December 2012, since the creation of the sculpted and profiled tail, flank tolerance seems to have been improved. Ureteral irritation in the region below the tail may be milder than that in the unmodified section. The thinning of the lower end of the device seems to limit the snagging of the stent during breathing movements (Fig. 1c). Industrial manufacturers will be required to produce such a device. The suture could then be integrated into the stent, emerging at the extreme end of the tail. In this way, stent tolerance might be improved.

We developed the PSS as a means of decreasing urinary symptoms, but we discovered fortuitously that it had other surprising properties, probably due to the simple presence of the sutures in the ureter.

**Fig. 3:**
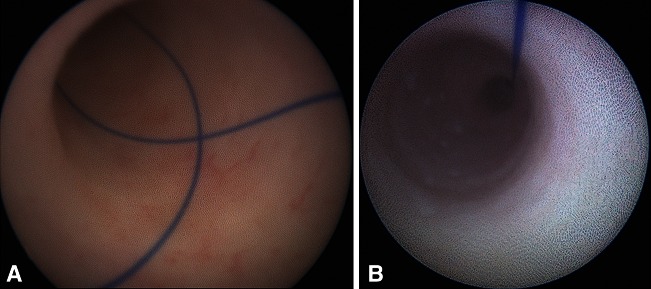
Dilation of the ureter 1 month after PSS implantation. **a** PSS sutures in the pelvic ureter. **b** PSS sutures in the lumbar ureter

**Fig. 4:**
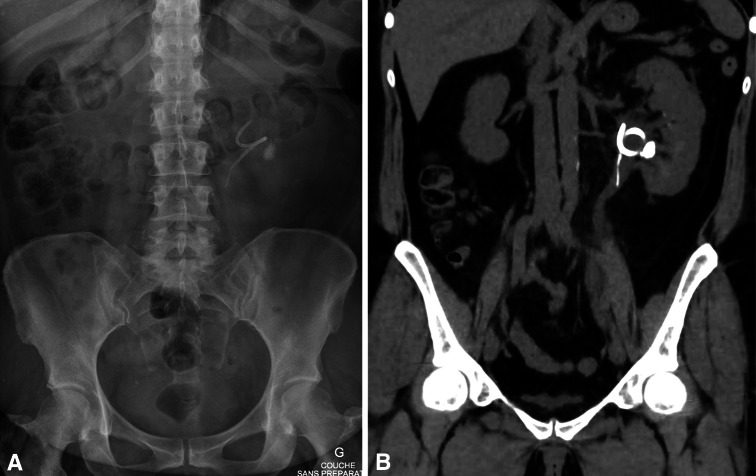
Patient with left renal stone and PSS. **a** Appearance of the PSS and the stone on X ray. **b** Dilated ureter on CT scan 1 month after PSS implantation

Firstly, about 1 month after PSS implantation, we observed in all cases of ureteroscopy clear dilation of the ureter intubated with the sutures (Fig. 3). It has been showed that preoperative stenting is effective for dilation of the ureter in preparation for ureteroscopy [21, 22] and insertion of an ureteral access sheath [22]. Three weeks seemed sufficient for dilation [21]. With the PSS, no patient required active dilation of the ureteral meatus at ureteroscopy. The prior implantation of a PSS could be used to prepare the ureter for the insertion of a sheath for flexible ureteroscopy without excessive discomfort. This could facilitate the introduction of a large ureteral access sheath (14/16F) for ureteroscopic treatment of large stone [23]. We believe that dilation was probably induced by the sutures. Ureteroscopy, fluoroscopic, and CT scan imaging allowed measuring the degree of dilation (Fig. 4). To clarify our endoscopic observations, we measured the diameters of the pelvis and the ureter drained by PSS and we compared them with the diameters of the same contralateral segments. Table 3 shows the results for 35 patients of this study with short PSS and only proximal stone.

**Table 3:** Diameter of the pelvis and the ureter on CT scan in patients with short PSS and proximal stone

	PSS side	Contralateral side	*p*
Number of CT scan analyzed	35		
Time before CT scan (days)	44.9 ± 28.0		
Right side	20		
Pelvis dilation	37.5 %		
Pelvis^a^	16.6 ± 6.9	6.6 ± 3.7	2 × 10^−6^
Upper lumbar ureter^a^	9.1 ± 1.8	3.1 ± 0.5	9 × 10^−8^
Lower lumbar ureter^b^	9.0 ± 2.3	3.6 ± 1.2	1 × 10^−13^
Iliac ureter^b^	7.2 ± 1.7	3.1 ± 0.6	8 × 10^−11^
Pelvic ureter^b^	6.4 ± 1.6	4.0 ± 0.8	1 × 10^−10^

The measurements of diameters are in mm

^a^Section with polyurethane stent alone

^b^Section with suture alone

**Fig. 5:**
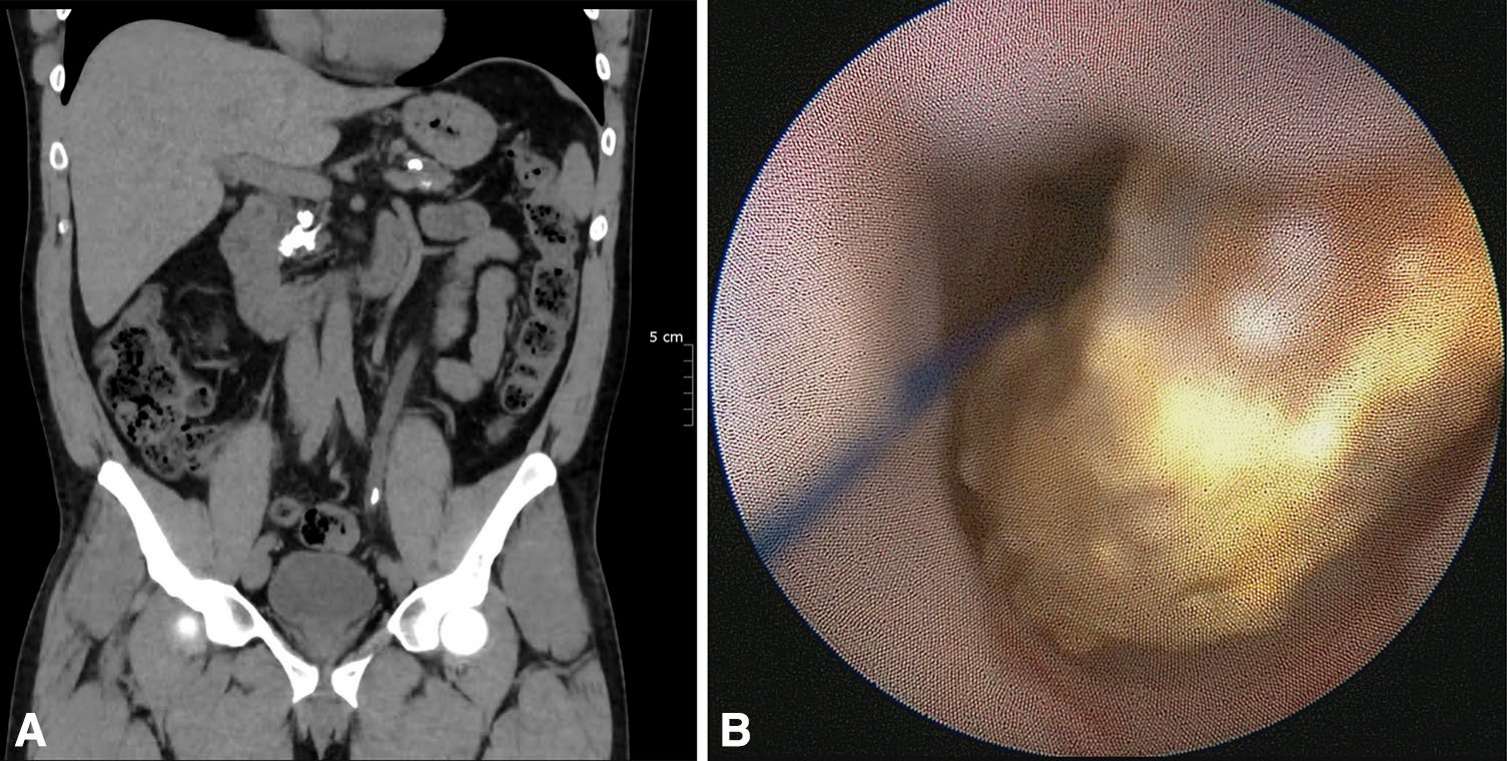
Patient with left ureteral stone and PSS. **a** Appearance of the stone along the suture in the dilated ureter on CT scan. **b** No ureteral inflammation is visible in contact with the stone (endoscopic appearance)

Secondly, after extracorporeal shockwave lithotripsy, the stone fragments gradually slid down the PSS sutures, without renal colic. Sutures behaves like a “stone’s toboggan” (Fig. 5). Ureteral dilation might accelerate the removal of stone fragments.

We believe that the use of a double-pigtail stent should no longer be considered the only way to drain the ureter. Instead, the form of the stent should depend on the patient’s disease. For example, in cases of non-obstructive kidney stones suitable for treatment by extracorporeal shockwave lithotripsy, we now use a stent reduced to a suture attached to a simple renal pigtail [4].

## Conclusion

The PSS significantly decreases urinary symptom and pain scores and constitutes a medical advance in the domain of ureteral stent tolerance. We observed unexpected dilation of the ureter by the sutures. We encourage and are convinced that multicenter studies with possibly a randomized, controlled trial would confirm the improvement in patient’s quality of life reported here. These studies would make it possible to enlarge the indications for the PSS and would also make it possible to investigate the other properties of the ureteral suture.

## Electronic supplementary material

Below is the link to the electronic supplementary material.

Supplementary material 1 (DOC 38 kb)

Supplementary material 2 (DOC 622 kb)

Supplementary material 3 (DOC 32 kb)

Supplementary material 4 (DOC 624 kb)

Supplementary material 5 (PDF 282 kb)
