# Supplementary material for: Changing the double-pigtail stent by a new suture stent to improve patient’s quality of life: a prospective study
Source: World J Urol. 2014 Sep 12;33(8):1061–8. doi: 10.1007/s00345-014-1394-2 (PMC4512273; doi:10.1007/s00345-014-1394-2)

**Docteur Benoît VOGT**

Ancien Interne des Hôpitaux de Paris

Ancien Chef de Clinique-Assistant des Hôpitaux de Paris

Ancien Chirurgien des Hôpitaux de Paris

——————

**Chirurgien Urologue**

**Polyclinique de Blois**

1, rue Robert Debré

41260 LA CHAUSSÉE SAINT-VICTOR

**Madame X**

Blois le *date*

Patient sonde JJ classique puis sonde nouvelle génération avec fil

Numéro d’identification anonyme : **X**

Madame,

Lors de votre intervention en chirurgie urologique, le *date* nous vous avons posé une sonde dans le rein.

Les sondes classiques appelées JJ sont généralement mal tolérées par les patients.

Aussi, à la Polyclinique nous avons mis au point un nouveau type de sonde afin d’améliorer la tolérance et éviter les symptômes douloureux.

Après avoir connu les sondes JJ classiques, vous avez donc reçu cette sonde nouvelle génération avec fil.

Nous envisageons un développement à grande échelle de ces sondes pour faire profiter encore plus de malades du confort de cette nouvelle technique.

Afin de vérifier la bonne tolérance de cette sonde, éventuellement l’améliorer encore, nous réalisons actuellement une enquête. **Nous souhaitons recueillir votre avis.**

En vous remerciant sincèrement de votre collaboration pour le bien de nos futurs patients.

Je vous prie de croire, Madame, à l’assurance de mes sentiments les meilleurs.

Docteur Benoît VOGT


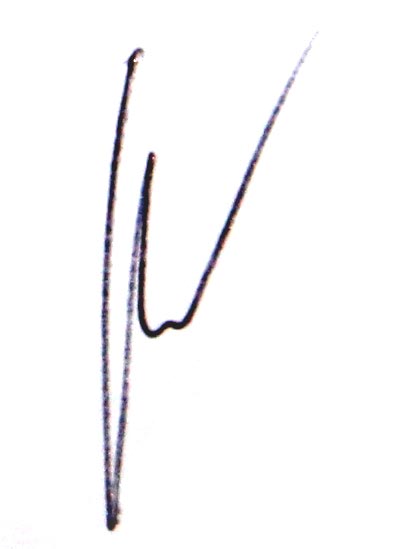

Supplement: Supplementary file 1 — Supplementary material 1 (DOC 38 kb) [file 345_2014_1394_MOESM1_ESM.doc]
