# Supplementary material for: Changing the double-pigtail stent by a new suture stent to improve patient’s quality of life: a prospective study
Source: World J Urol. 2014 Sep 12;33(8):1061–8. doi: 10.1007/s00345-014-1394-2 (PMC4512273; doi:10.1007/s00345-014-1394-2)
Supplement: Supplementary file 3 — Supplementary material 3 (DOC 32 kb) [file 345_2014_1394_MOESM3_ESM.doc]

**Questionnaire sur vos signes urinaires habituels**

**Nous vous remercions pour votre participation - Toutes ces informations resteront confidentielles.**

Identifiant anonyme :

**Age** : ……. ans **Sexe** : Femme  Homme  **Poids**: …… kg **Taille**: …… cm

**1 – Vos symptômes urinaires habituels**

🡺 Ne cocher qu’une seule case par question.

**1. Pendant la journée, à quelle fréquence allez-vous uriner, en moyenne ?**

Plusieurs fois par heure  Toutes les 3 heures  Toutes les 4 heures ou plus 

Toutes les heures  Toutes les 2 heures 

**2. Pendant la nuit, combien de fois vous levez-vous pour aller uriner, en moyenne ?**

Aucune  1 fois  2 fois  3 fois  4 fois ou plus 

**3. Avez-vous besoin de vous précipiter aux toilettes pour uriner ?**

Jamais  Rarement  Parfois  La plupart du temps  Tout le temps 

**4. Avez-vous des fuites d’urine avant de pouvoir vous rendre aux toilettes ?**

Jamais  Rarement  Parfois  La plupart du temps  Tout le temps 

**5. Avez-vous des fuites d'urine sans ressentir le besoin d'aller uriner ?**

Jamais  Rarement  Parfois  La plupart du temps  Tout le temps 

**6. Avez-vous la sensation que votre vessie ne se vide pas correctement après avoir uriné ?**

Jamais  Rarement  Parfois  La plupart du temps  Tout le temps 

**7. Ressentez-vous une sensation de brûlure lorsque vous urinez ?**

Jamais  Rarement  Parfois  La plupart du temps  Tout le temps 

**8. A quelle fréquence observez-vous du sang dans vos urines ?**

Jamais  Rarement  Parfois  La plupart du temps  Tout le temps 

**9. Quelle quantité de sang observez-vous dans vos urines ?**

Pas de sang  Urine légèrement teintée  Urine fortement teintée  Urine teintée et caillots 

**10. Vos symptômes urinaires représentent-ils un problème pour vous ?**

Pas du tout  Un peu  Modérément  Beaucoup  Extrêmement 

**11. Si vous deviez vivre le restant de votre vie avec ces symptômes urinaires, diriez-vous que vous en seriez:**

Très satisfait  Satisfait  Plutôt satisfait  Partagé  Plutôt ennuyé  Ennuyé  Très ennuyé 

**2 – Les douleurs ressenties**

**12. Ressentez-vous habituellement des douleurs ?**

OUI  NON 

➀– Dos ➁- Flanc ➂- Bas ventre ➃ - Pénis (pour les hommes)

Pas de douleur ni gêne Pire douleur possible
