# Supplementary material for: Changing the double-pigtail stent by a new suture stent to improve patient’s quality of life: a prospective study
Source: World J Urol. 2014 Sep 12;33(8):1061–8. doi: 10.1007/s00345-014-1394-2 (PMC4512273; doi:10.1007/s00345-014-1394-2)
Supplement: Supplementary file 4 — Supplementary material 4 (DOC 624 kb) [file 345_2014_1394_MOESM4_ESM.doc]

**Questionnaire de tolérance de la sonde nouvelle génération avec fil**

**Nous vous remercions pour votre participation - Toutes ces informations resteront confidentielles.**

Identifiant anonyme :

**Age** : ……. ans **Sexe** : Femme  Homme  **Poids**: …… kg **Taille**: …… cm

**Date de pose de votre sonde : ……/……/20…… Coté** : gauche  droit 

**1 – Vos symptômes urinaires après la pose de la sonde nouvelle génération avec fil**

🡺 Ne cocher qu’une seule case par question.

**1. Depuis la pose de la sonde nouvelle génération avec fil, pendant la journée, à quelle fréquence allez-vous uriner, en moyenne ?**

Plusieurs fois par heure  Toutes les 3 heures  Toutes les 4 heures ou plus 

Toutes les heures  Toutes les 2 heures 

**2. Pendant la nuit, combien de fois vous levez-vous pour aller uriner, en moyenne ?**

Aucune  1 fois  2 fois  3 fois  4 fois ou plus 

**3. Avez-vous besoin de vous précipiter aux toilettes pour uriner ?**

Jamais Rarement Parfois La plupart du temps  Tout le temps

**4. Avez-vous des fuites d’urine avant de pouvoir vous rendre aux toilettes ?**

Jamais Rarement Parfois La plupart du temps  Tout le temps

**5. Avez-vous des fuites d'urine sans ressentir le besoin d'aller uriner ?**

Jamais Rarement Parfois La plupart du temps  Tout le temps

**6. Avez-vous la sensation que votre vessie ne se vide pas correctement après avoir uriné ?**

Jamais Rarement Parfois La plupart du temps  Tout le temps

**7. Ressentez-vous une sensation de brûlure lorsque vous urinez ?**

Jamais Rarement Parfois La plupart du temps  Tout le temps

**8. A quelle fréquence observez-vous du sang dans vos urines ?**

Jamais Rarement Parfois La plupart du temps  Tout le temps

**9. Quelle quantité de sang observez-vous dans vos urines ?**

Pas de sang Urine légèrement teintée Urine fortement teintée Urine teintée et caillots

**10. Depuis la pose de la sonde nouvelle génération avec fil, vos symptômes urinaires représentent-ils un problème pour vous ?**

Pas du tout Un peu  Modérément  Beaucoup  Extrêmement 

**11. Si vous deviez vivre le restant de votre vie avec ces symptômes urinaires, diriez-vous que vous en seriez:**

Très satisfait Satisfait  Plutôt satisfait  Partagé Plutôt ennuyé Ennuyé Très ennuyé

**2 – Les douleurs ressenties suite à la pose de la sonde nouvelle génération avec fil**

**12. Ressentez-vous des douleurs ou une gêne suite à la pose de la sonde nouvelle génération avec fil ?**

 OUI passez à la question 13  NON passez à la **rubrique 3**

**13. Depuis l’intervention, ressentez-vous des douleurs ou une gêne au niveau des reins lorsque vous urinez ?**

Non Oui

**Tournez la page**

**14. À quelle fréquence avez-vous besoin de médicaments anti-douleur pour calmer la douleur ou la gêne liées à la sonde nouvelle génération avec fil?**

Jamais Rarement Parfois La plupart du temps  Tout le temps

**15. Les dessins ci-dessous représentent votre corps.**

Marquez avec une croix (X) ou colorez le (ou les endroits) où vous éprouvez des douleurs ou une gêne en rapport avec la sonde nouvelle génération


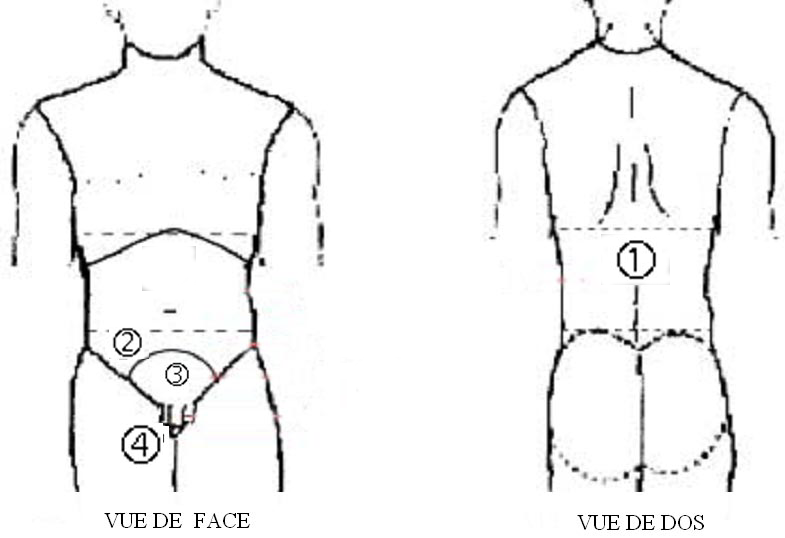


Les chiffres  –  représentent les zones suivantes pour les cotés droit et gauche :

– Dos - Flanc - Bas ventre  - Pénis (pour les hommes)

Merci de dessiner un O pour toute autre aire non marquée et écrire le nom de ce site.

**16. Placer une croix (X) sur la ligne ci-dessous pour représenter vos douleurs ou gêne en rapport avec la sonde nouvelle génération avec fil.**

Si la douleur est présente sur plusieurs sites avec une intensité différente, merci d’utiliser des marques différentes en utilisant les mêmes chiffres que sur l’image ci-dessus pour les localiser.

Pas de douleur ni gêne Pire douleur possible

**3 – Les incidences de la sonde nouvelle génération avec fil sur votre qualité de vie, votre travail ou vos activités**

**17. Les gênes ou les douleurs survenues suite à la pose de la sonde nouvelle génération avec fil entraînent-elles des arrêts de travail ou un ralentissement de votre activité ?**

Jamais Rarement Parfois  La plupart du temps  Tout le temps

**Si vous avez des commentaires sur le questionnaire ou si vous voulez ajouter des précisions sur les symptômes liées à la sone nouvelle génération avec fil, merci d'utiliser l'espace ci-dessous.**

…………………………………………………………………………………………………………………………………………………………………………………………………………………………………………………………………………………………………………………………………………………………………………………………………………………………………………………………………………………………………………
