# Supplementary material for: Changing the double-pigtail stent by a new suture stent to improve patient’s quality of life: a prospective study
Source: World J Urol. 2014 Sep 12;33(8):1061–8. doi: 10.1007/s00345-014-1394-2 (PMC4512273; doi:10.1007/s00345-014-1394-2)
Supplement: Supplementary file 5 — Supplementary material 5 (PDF 282 kb) [file 345_2014_1394_MOESM5_ESM.pdf]

Comité de Protection des Personnes
« Ile de France II »

IRB registration # : 00001072

Bureau :
Présidente : Marie-France MAMZER-BRUNEEL
Vice-Président : Christian HERVE
Secrétaires : Pierre COLONNA, Gérard PELE
Trésorier : Stéphane DONNADIEU
Membres :
C. ARDIOT, J.-L. BRESSON, C. BROISSAND, J.-B. CHARPENTIER, G. CHATELLIER, P. COLONNA, B. DEBAECKER, N. DELSARTE, S. DONNADIEU, J. FAGARD, C. HERVE, L. LEBOUCHER, A. LEVY, M.-F. MAMZER-BRUNEEL, E. MARTINENT, O. PARENT de CURZON, M. PARISOT, G. PELE, R. QUERE, G. QUEVA, C. RAMBAUD, M.-C. REINMUND, M. RUDLER, P. VAN ES, G. VOGT
Secrétariat : Guillaume VOGT, Marie France MAMZER

PARIS, February 13th 2014

Ref. 2014-02-02

The French Ethical Committee, Comité de Protection des Personnes (CPP), Ile de France 2 (IDF2) **has approved** the submission of your project study entitled:

"Pigtail Suture Stent: Decisive Progress Towards Double-Pigtail Stent Tolerance. A Prospective Study."

Principal Investigator : **Docteur Benoît VOGT**,
Former intern at the Paris Hospitals
Former Assistant Chief of Clinic-Hospital of Paris
Former Surgeon Hospitals of Paris
Surgeon Urologist
Polyclinique de Blois
1, rue Robert Debré
41260 LA CHAUSSÉE SAINT-VICTOR

Secrétariat : 02 54 90 65 60
Cell : 06 63 22 08 44
Email: benoitvogt@free.fr

This study has received the approval for Initial Review

This approval is based on its design that permit to consider it as a « study on current cares » according to the French law (Art. L 1121-1). In this study, the risks are insignificant and the benefits are those of the standard care.

IRB APPROVAL

Dr Marie-France MAMZER-BRUNEEL,
Présidente du CPP IDF II

Centre Universitaire des Saints-Pères, 45 rue des Saints-Pères, 75006 PARIS
6ème étage, Porte 621 Tél. 01 42 86 41 34 - Fax 01 42 86 41 33 - Courriel : cpp.iledefrance2@yahoo.fr
